# Sentence production in rehabilitation of agrammatism: A case study

**DOI:** 10.1590/S1980-57642014DN83000015

**Published:** 2014

**Authors:** Marcela Lima Silagi, Fernanda Naito Hirata, Lúcia Iracema Zanotto de Mendonça

**Affiliations:** 1 Speech Pathologist, MSc, Department of Physiotherapy, Speech-Language and Hearing Sciences and Occupational Therapy, School of Medicine, University of São Paulo, SP, Brazil.; 2 Speech Pathologist. Laboratory of Neurolinguistic Investigation, School of Medicine, University of São Paulo, SP, Brazil.; 3 Neurologist, MD, PhD – Clinicas Hospital – School of Medicine, University of São Paulo, SP, Brazil. Catholic University of São Paulo, SP, Brazil.

**Keywords:** agrammatism, aphasia, rehabilitation

## Abstract

Agrammatism is characterized by morphosyntactic deficits in production of sentences. Studies dealing with the treatment of these deficits are scarce and their results controversial. The present study describes the rehabilitation of a case diagnosed as chronic Broca's aphasia, with agrammatism, using a method directed to sentence structural deficits. The method aims to expand the grammatical repertoire by training production of sentences with support from contexts that stimulate actions and dialogues. The patient showed positive results on all types of sentences trained and generalized the gains to spontaneous speech. However, these benefits were not sustained in the long term.

## Abstract (pt)

O agramatismo é marcado por déficits morfossintáticos na produção das sentenças. Estudos que abordam o tratamento destas dificuldades são escassos e controversos. O presente estudo descreveu a reabilitação de uma paciente com agramatismo associado à afasia de Broca em fase crônica, por meio de programa de estruturação de sentenças. Trata-se de método que visa à expansão do repertório gramatical através do treino com apoio de contextos que estimulam ações e diálogos. A paciente obteve resultados positivos em todos os tipos de frases treinadas, com ganhos generalizados para contextos de fala espontânea. No entanto, os benefícios não se mantiveram em longo termo.

## INTRODUCTION

Broca's aphasia can result from focal cerebrovascular accident or larger lesions beyond the Broca's area.^1^ This syndrome is characterized by reduced fluency with impaired repetition of sentences, difficulties in naming, reading and writing with relatively preserved comprehension of language. Agrammatism with sentences that are reduced in both length and grammatical complexity contribute to reduced fluency.^2^ The speech of individuals with agrammatism is paused and characterized by omission/substitution of grammatical morphemes and verbs.^3^ Besides spontaneous speech, agrammatism can be observed in repetition, reading and writing. The oral comprehension of sentences whose meanings depend on the syntax can also be affected.^2,3^ Caplan^4^ interprets agrammatic deficits as a result of a reduction in cognitive resources such as working memory and attention.

Agrammatism is not expressed homogeneously in Broca's aphasia. Faroqi-Shah and Thompson^5^ report that some individuals have deficits predominantly in verb production, while others have difficulty using items from closed grammatical classes. Differences in manifestation across languages can also be expected. Among the different approaches to rehabilitation, some methods focus on structuring sentences; others focus on the production of verbs while a third centers on the use of functional grammatical categories.^5^ Wisenburn et al.^6^ conducted a meta-analysis of 21 studies on the efficacy and effectiveness of therapy for agrammatism and found gains in most approaches. One of the methods for the treatment of agrammatism described in the literature is the Sentence Production Program for Aphasia (SPPA).^7^ The method aims is to expand the repertoire of grammatical structure of sentences. The sentence-stimuli were selected from the observation of frequent errors among persons with aphasia.^8^ The training is hierarchized from repetition to spontaneous production in context. SPPA is indicated for individuals with basic preservation of auditory comprehension, memory and attention. The SPPA was translated from its original English version into Brazilian Portuguese, without adaptations. A consensus on the Brazilian version (was not published) was obtained after exhaustive discussion.

Thompson and Bastiaanse^9^ argue that the study of agrammatism is important both theoretically and clinically, because it comprises important domains of data that can serve as a test for models of normal linguistic capacity and plays a crucial role in the construction of these models. Moreover, studying patterns of recovery from agrammatism can also be informative, providing a window into the organization of the language system. The same authors stated that lesion-deficit studies also remain important as a means of relating linguistic constructs to the brain mechanisms of language, specifically issues associated to neuroplasticity.

This case-study is of interest for the following reasons:

[A] the heterogeneity of patients with agrammatism which hampers the establishing of a homogeneous sample and justifies single-case studies;
[B] the lack of information about agrammatism, particularly in Portuguese;
[C] the scarcity of studies on rehabilitation of agrammatic patients with large left hemispheric lesions - most studies focus on mild-moderate impairments;
[D] the controversies regarding the therapeutic results obtained using the method of structuring sentences. Against this background, the aim of the present study was to describe the rehabilitation of a patient with agrammatism associated with Broca's aphasia, using a method of structuring sentences.

## CASE REPORT

**Figure 1:**
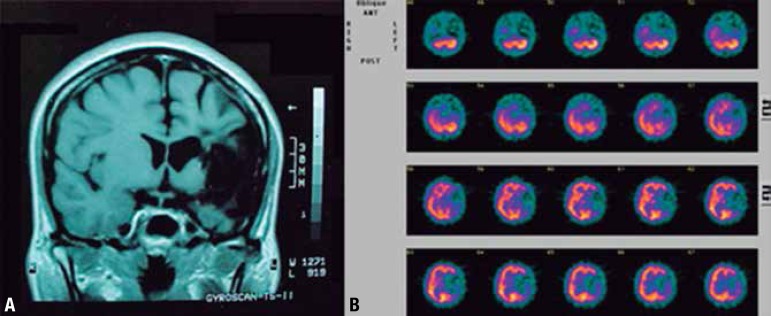
[A] Magnetic Resonance Imaging (MRI). Sequela affecting the cortex in left fronto-temporo-parietal region, centrum semiovale and also the subcortical caudate nucleus. The affected structures were the inferior frontal gyrus, insula, superior temporal gyrus and inferior parietal region. Ectasia ex-vacuo was detected in the left lateral ventricle. [B] Single Photon Emission Computed Tomography (SPECT)*. Marked perfusion deficits in left frontal, temporal and inferior parietal regions and moderately high deficit in the left parietal region.

GAA, a 35-year-old woman with 10 years of schooling, dextral, and retired packer, suffered an ischemic cerebrovascular accident (CVA) in the region of the middle cerebral artery (Figure 1) in 1995.

Magnetic resonance imaging (MRI), (24/10/2001) revealed lesions in cortical and subcortical areas of language (Figure 1A). On Single Photon Emission Computed Tomography (SPECT) (31/10/2001) (Figure 1), there was a large perfusion deficit in the left hemisphere (Figure 1B). Besides global aphasia syndrome, diagnosed in the acute phase and which evolved to Broca's aphasia with severe agrammatism, GAA had persistent right hemiplegia, predominantly in the upper limb. Ten years had elapsed between the CVA and the reported therapeutic intervention.

**Procedures.** Before training production of sentences with the SPPA,^7^ GAA was evaluated by the Boston Diagnostic Aphasia Examination-Short Form (BDAE-SF) which includes sub-tests of oral comprehension of words, sentences and texts, oral confrontation naming, repetition, production of descriptive (The Cookie Theft scene) and written language.^3^ These data were registered on videos. An additional description of the Cookie Theft scene was used as an independent measure to analyze generalization and transfer of learning. Severity of aphasia was rated by the Boston Aphasia Severity Rating Scale, a measure used as a summary of sub-tests. Previously, she was submitted to a conventional multimodal stimulation therapy^10^ to elicit language production through repetition, phonemic cueing, reading in a variety of linguistic and situational contexts, and consequently improved repetition of short phrases and global communication (initiative to introduction, maintenance and diversity of topics, increasing of partners of conversation).

On the SPPA, eight types of sentence were trained. Each type contained 15 stimuli (total of 120 figures of scenes+ target sentences). Each type of sentence was presented at two levels of difficulty, as prescribed by the method: 1) Level A - the target sentence was presented simultaneously with an action scene, depicting its use in context, for repetition after the speech therapist had presented the story. 2) Level B - the story had to be complemented with the target sentence, without the benefit of repetition. (See examples online - Figure 3).

The sentences of each type were presented in blocks. When GAA answered correctly at level A (facilitation by repetition), the stimulus level B (confrontation of scene for spontaneous emission of the target sentence) was immediately presented. The SPPA was planned for 32 sessions distributed in weekly sessions of 30 minutes (about four sessions per sentence).

Performance was scored according to the following criteria: 1 point for correct answers (including successful self-correction, after initial error); 0.5 for partially correct answers (only one word was omitted or mistakenly produced, compromising both the syntax and the meaning of the sentence); 0 for incorrect responses in which two or more words were omitted or incorrectly produced, or if the target sentence contained only one word and it was not produced correctly. Situations when GAA failed at level A and where consequently level B was not applied, were considered "not applicable" (NA). The criterion for the continuation of the blocks was a score of at least 13 out of 15 items (85% correct).

## RESULTS

On the BDAE-SF reduced version, applied pre-SPPA, the patient showed functional oral language comprehension, non-fluent, reduced oral expression, restricted to single words with pauses, prolongations and occasionally phonemic paraphasias. Oral language comprehension pre-SPPA was: 15/16 (93%) for comprehension of words; 9/10 (90%) in comprehension of commands; 3/6 (50%) in auditory comprehension of complex material.

On the Aphasia Severity Rating Scale, communication ability was "Through fragmentary expression; great need for inference, questioning, and guessing by the listener. The range of information that can be exchanged is limited, and the listener carries the burden of communication, which corresponds to level 1 (minimum score = 0, maximum = 5).

The profile of the patient was consistent with the diagnosis of Broca's aphasia with agrammatism. Results of BDAE-SF pre-therapy, post-therapy and follow-up after one year are available in Table 1 on-line.

**Figure 2:**
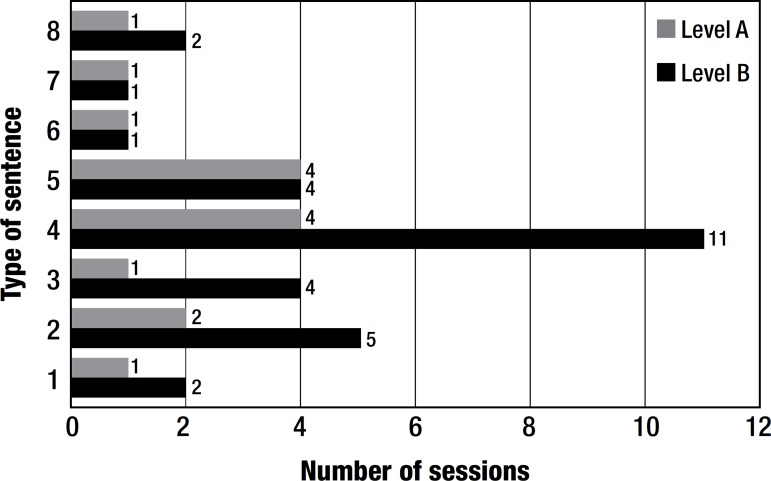
Number of sessions needed to attain 85% correct answers.

GAA underwent 30 weekly sessions of SPPA. Her performance is depicted in Figure 2. She did not practice the SPPA at home, and was encouraged to engage in conversations based on daily events.

GAA improved performance after training with the SPPA. She was able to repeat, and spontaneously build, all types of sentences although some kinds of sentences proved more difficult. The horizontal sequence shows the number of sessions designed for blocks of each type of sentence. Qualitative performance of the patient and number of sessions are available on-line - Figure 4.

After the SPPA, GAA was able not only to expand the type of sentences produced but also the use of verbs and function words for a semi-spontaneous situation - description of the Cookie Theft scene (Figure 5 on-line).

## DISCUSSION

This study described the rehabilitation of a patient with agrammatism associated with Broca's aphasia, by a method of structuring sentences, the SPPA. The immediate results were positive and the patient was able to build all types of sentences after 32 sessions of therapy. These gains were transferred to semi-spontaneous production elicited by the Cookie Theft scene.^3^ The positive results obtained immediately after training, however, need to be examined with caution.

GAA was in the chronic phase of CVA at the beginning of training with the SPPA. The aphasia was severe, with production limited to telegraphic sentences, absence of verbs and functional morphemes. Previous therapeutic trials had not reduced the agrammatism. In training with the SPPA, despite having difficulties with verbs, GAA required about 3.75 sessions to achieve spontaneous production in most types of sentences, which is compatible with the expectations of the author.^7^ It is possible that the simultaneous visual confrontation of the action verbs in the scenes facilitated the performance of GAA. Positive results in therapy to produce verbs from observation of videos have been reported.^11^ Although this strategy has not been applied to GAA, it is possible that the observation of static actions in context triggered representations of actions, leading to beneficial results, similar to the effect of the videos. However, in more complex situations such as transformations of syntax (canonical to interrogative), GAA had greater difficulty producing the sentence spontaneously, without the support of repetition. This was the case of interrogative sentences built with morphemes of time (when) and place (where). GAA omitted the morphemes and adopted the telegraphic style. In addition to processing the "interrogation", she had to employ adverbial forms which refer to verbs, a difficult category to produce for the patient. GAA needed 11 sessions to produce this type of sentence spontaneously.

In the description of the Cookie Theft scene, which was not trained, GAA improved the use of function words, particularly articles, and decreased agrammatic deletions. These positive gains immediately after stimulation by SPPA however, were not maintained at follow-up after a year.

There are controversies over positive results using the method of structuring sentences, SPPA, in the treatment of agrammatism. Helm-Estabrooks and Nicholas^7^ reported positive results, Doyle^12^ et al and Fink^13^ verified partially positive results with restriction of gains to the type of sentences trained while Faroqi-Shah and Thompson proposed that partial results are due in part to the severity of the aphasia.^5^ The results of GAA did not support this position. After the SPPA, gains were observed in language production in untrained situations, such as the description of the Cookie Theft scene. Furthermore, GAA increased the use of grammatical morphemes from training structuring of sentences, which constitutes an inter-modality transfer.

We must consider that GAA had severe aphasia, which may have contributed to the absence of positive results in the follow-up. Another important factor to be discussed is the time elapsed between the stroke and application of SPPA, beyond the desirable range recommended by the literature. Moreover, we must consider that the effects of intensive and concentrated practice, valued in the literature on aphasia rehabilitation, were not taken into account in this therapeutic program.^14-16^

The structural (MRI) and functional (SPECT) neuroimages of GAA revealed sequela in the left hemisphere, including basal ganglia, in parallel with integrity of the right hemisphere. The large lesion of GAA compromises the areas previewed in a cortical model for agrammatism.^9^ Recent studies, based on a lesion model, have demonstrated the role of striatal damage in grammar learning and use. Observations of Huntington's disease in its early stage have provided data about morphological and syntactic difficulties.^17,18^ The extent of GAA's lesion precludes the justification of gains by recruitment of areas of the left hemisphere; one possible explanation is recruitment of the right hemisphere. The use of sentences in context, as proposed by SPPA, induces activation of semantic aspects of syntactic production in the preserved hemisphere. The recruitment of homologous areas of the right hemisphere to compensate for syntactic deficits was shown in tests using the functional technique after CVA-induced aggramatism^19^ and in cases of CVA.^20^

Considering the study design and the effects of rehabilitation, it is possible to classify the evidence from this case study for clinical practice under class III, and recommend it as optional, with possible effectiveness in practice in rehabilitation of agrammatism.^21^ Recent literature on efficacy of aphasia recognizes the positive effect of an intensive and concentrated training schedule, but these studies are limited to lexical deficits.^14-16^ An interesting perspective would be to verify the effects of massive and concentrated practice on structuring sentences, as well as parsing of underlying forms of sentences and training of verbs and other morpho-functional categories.

In conclusion, it was possible to describe and verify partially positive results in this case study with the SPPA. In severe cases such as that of GAA, it is interesting to take into account the possibility of using combined methods, structured based on the principles of evidenced-based practice.
